# Editorial: Psychological therapies for the management of chronic pain

**DOI:** 10.3389/fpain.2023.1219971

**Published:** 2023-06-16

**Authors:** Mery Paroli, Giovane Galdino

**Affiliations:** ^1^ Anaesthesiology and Pain Therapy Unit, Santa Chiara University Hospital, Pisa, Italy; ^2^ Pain Neuroimmunobiology Laboratory, Institute of Motricity Sciences, Federal University of Alfenas, Alfenas, Brazil

**Keywords:** pain, psychological, non-pharmacological treatments, research pain, pathology

**Editorial on the Research Topic** Psychological therapies for the management of chronic pain

Pain is a significant global health problem affecting millions of people worldwide. According to the World Health Organization (WHO), one in five adults worldwide suffers from moderate to severe chronic pain, and this number increases to one in three for people over the age of 65 (1). The prevalence of chronic pain is higher in low- and middle-income countries, and it is estimated that chronic pain affects over 60% of adults in some countries (2).

Psychological therapies have emerged as effective treatment options for managing chronic pain. These therapies have been shown to be effective non-pharmacological treatments for managing chronic pain; in addition, they help to improve the quality of life of patients by controlling the psychological and emotional aspects of chronic pain.

Over the years, studies have increasingly confirmed that pain does not only involve the physical component, in which its treatment depends on the improvement and psychological well-being of the patient. Thus, the studies addressed this topic involved investigating acceptance of pain, comparing gender differences in pain, evaluating the effect of pain awareness via telehealth, and comparing non-pharmacological interventions such as Yoga and structured exercise.

In the first study, conducted by Allsop et al., the authors conducted a clinical trial with veterans with fibromyalgia, in which they compared Yoga vs. Structured Exercise (moderate-intensity aerobic and strength training exercises) on pain intensity. Both interventions consisted of 1-month 75-minute classes (early response), 3 months (immediate post-intervention), and 6 and 9 months (sustained effects). In addition to pain intensity was also assessed the Brief Pain Inventory (BPI) as the primary outcome, and fatigue, sleep quality, physical function, and quality of life as secondary outcomes. In addition to being the first study to compare yoga-based intervention to a standardized exercise intervention that includes strength and aerobic training for patients with fibromyalgia, it presented a focus on the interplay among biological, psychological, and social factors that are important outcomes to be investigated by non-pharmacological treatments for pain control.

In another study on this topic (Windgassen et al.), the difference in the experience of interstitial cystitis/bladder pain syndrome (IC/BPS) between men and women was evaluated. IC/BPS is a chronic condition characterized by bladder pain and urinary frequency, and it is more common in women than in men. The study found that women with IC/BPS experience more severe pain, urinary symptoms, and sexual dysfunction than men. Women also report higher levels of anxiety and depression related to IC/BPS. The authors highlight the importance of recognizing gender differences in the experience of IC/BPS and tailoring treatment approaches accordingly. They suggest that future studies should focus on understanding the biological, psychological, and social factors that contribute to gender differences in IC/BPS.

The third and interesting study of this special tissue evaluated the effect of a single-session, telehealth emotional awareness and expression therapy (EAET) class on reducing pain, stress, and negative emotions in patients with chronic pain (Ziadni et al.). The results showed that this intervention was feasible and acceptable to patients with chronic pain. The participants reported a significant reduction in pain intensity, pain interference, stress, and negative emotions immediately after the intervention, and the effects were sustained at a 30-day follow-up. Despite the important results, further research with a larger sample size and a randomized controlled design is needed to confirm these findings and establish the effectiveness of this intervention.

The last study investigated the predictive power of acceptance and coping on adjustment to endometriosis (Bernini et al.). It demonstrated that both acceptance and coping strategies were significant predictors of adjustment to endometriosis. However, acceptance had incremental validity over coping in predicting better adjustment to the condition, even when controlling for other factors such as pain severity, age, and duration of the condition. Thus, this work may contribute to the growing body of literature on the importance of acceptance in chronic illness management. It suggests that acceptance may be more effective than coping strategies in helping women with endometriosis adjust to their condition and cope with the challenges associated with it.

In conclusion, this topic demonstrates the importance of education about the pathology, in addition to the use of both assessment techniques that focus on the influence of psychological factors that may be involved in chronic pain and the effectiveness of non-pharmacological treatments such as physical activity that may help to improve pain mental health and pain management.
